# A protocol for systematic review of *Plantago major* L. effectiveness in accelerating wound-healing in animal models

**DOI:** 10.1186/s13643-019-1255-6

**Published:** 2019-12-23

**Authors:** Fernanda de Cássia Israel Cardoso, Priscila Peruzzo Apolinário, Jéssica da Silva Cunha Breder, Thalita Paranhos, Henrique Ceretta Oliveira, Ariane Dini Polidoro, Ana Railka Souza Oliveira Kumakura, Maria Helena Melo Lima

**Affiliations:** School of Nursing, State University of Campinas (UNICAMP), Rua Tessália Vieira de Camargo, 126, Campinas, SP – CEP 13083-887 Brazil

**Keywords:** Plantaginaceae, Systematic review, Wound healing, Wounds and injuries

## Abstract

**Background:**

Studies have indicated that *Plantago major* L. (*P. major*) has therapeutic properties, such as anti-inflammatory, antioxidant, antifungal, immunostimulatory, and tissue regeneration. This plant species is assumed to provide potent tissue repair and healing in treatments of skin wound injuries, but the understanding of its effectiveness is still unclear. The systematic review proposed herein aims to assess effectiveness of *P. major* for wound healing in animal models.

**Methods:**

We will conduct database searches in BVS, PubMed, Scopus, Web of Science, CINAHL, and CABDirect. Reviewers will independently evaluate titles, abstracts, and full-text articles retrieved from databases to identify potentially eligible studies. Relevant articles will be assessed for risk of bias and quality. The database searches will include analysis of wound healing rate through macroscopic evaluation, photo images, or calculation of the wound area retraction until the wound closure. Relevant data will be compiled for the capability and effectiveness of *P. major* treatments in accelerating wound healing. Random effects meta-analysis models will be employed to compare among groups based on outcome variables from studies reporting sufficient high-quality data.

**Discussion:**

Results of this systematic review will be presented in a narrative synthesis form. They will provide a summary and clear understanding of the relevant current questions and evidences directly related to *P. major* effective tissue repair and healing. Outcomes of this systematic review will contribute with important information that could benefit future research efforts and potential applicability in humans.

**Systematic review registration:**

PROSPERO CRD42019121962

## Background

Because of their therapeutic effects, medicinal plants are used worldwide to treat many diseases. They are rich sources of phytochemicals with potential therapeutic effect in treatments using direct application of raw material. Moreover, they also play a role for the development of new medicinal drugs. *Plantago major* L. is one of the most abundant and widely distributed medicinal plant in the world. It is a perennial plant species that belongs to the genus *Plantago* and the family Plantaginaceae [1]. *P. major* leaves and seeds are reported to have analgesic, anti-inflammatory, antioxidant, immunomodulatory, antifungal, anticancer, and wound healing [2–4]. Medicinal benefits of *P. major* may be related to various bioactive compounds, such as flavonoids, alkaloids, terpenoids, phenolic compounds, iridoid glycosides, fatty acids, polysaccharides, and vitamins [2, 5]. Recent studies have shown successful treatment of cutaneous wounds with certain plant species or natural substances isolated from plants [6–8]. *P. major* has been described to be effective for tissue repair and skin wound healing, but the extent of its effectiveness has not been evaluated.

First reports related to therapeutic uses of this plant species date from the twelfth and thirteenth centuries [9, 10]. Nowadays, several researches using in vivo, in vitro, and ex vivo techniques have demonstrated potential healing activity of ethanol- and water-based extracts from *P. major* leaves and seeds [11–14]. To date, a systematic review on therapeutic effectiveness of this plant species on skin wound healing was not identified in the literature. Therefore, the findings of this proposed systematic review will significantly contribute to the current knowledge of the mechanism of action of *P. major* in the process of cutaneous wound healing.

According to the literature, *P. major* has various medicinal applications without any serious adverse effects. Besides, this plant species is widely distributed in many countries (11-14). These evidences encourage further search on the effectiveness of *P. major* on healing processes, as well as transfer of knowledge from research to clinical practice. Thus, the objective of this systematic review is to identify, select, and evaluate high-quality published research on the effectiveness of *P. major* in the healing process of cutaneous wounds.

### Study question

What is the evidence in the literature for the effectiveness of using *P. major* for wound healing in animal models?

## Methods

### Protocol and registration

This protocol was registered on PROSPERO (CRD42019121962). The bias of the assessed experimental studies will be evaluated by the Systematic Review Center for Laboratory Animal Experimentation (SYRCLES) [15]. The quality of the studies will be evaluated by the Collaborative Approach to Meta-Analysis and Review of Animal Data from Experimental Studies (CAMARADES) [16]. This systematic review will search for primary studies in animal models with cutaneous wounds topically treated with *P. major* in comparison with the placebo/vehicle control group.

### Electronic search methods for study identification

Search will be conducted in 7 electronic bases listed below alongside with their respective strategies: *BVS*: (“Wound Healing”) OR (Regeneration) AND (“Plantago major” OR “Plantago officinarum”). *PubMed*: (Wound Healing) OR “Wound Healing”) OR Healing, Wound) OR “Healing, Wound” OR Healings, Wound OR “Healings, Wound”) OR Wound Healings) OR “Wound Healings”) OR (Regeneration) OR Regeneration) OR Regenerations) OR Regenerations) AND (“Plantago major”) OR “Plantago officinarum”). *Scopus*: (“Wound Healing” OR “Healing, Wound” OR “Healings, Wound” OR “Wound Healings”) OR (TITLE-ABS-KEY (regeneration OR regenerations) AND (TITLE-ABS-KEY (“Plantago major” OR “Plantago officinarum”). *Web of Science*: (“Wound Healing” OR “Healing, Wound” OR “Healings, Wound” OR “Wound Healings”) (Regeneration OR Regenerations) (“Plantago major” OR “Plantago officinarum”). *Embase*: (“plantago major” OR “plantago major” OR “plantago major”) AND (“wound healing”/exp OR “wound healing”/syn OR “wound healing” OR “regeneration”/ OR “regeneration” OR “regeneration”). *CINAHL*: (“Wound Healing”) OR “Wound Healing” OR “Healing, Wound” OR “Healings, Wound” OR “Wound Healings” OR (“Regeneration”) OR Regeneration OR Regenerations AND “Plantago major” OR “Plantago major” OR “Plantago officinarum”. *CABDirect*: (Regeneration OR Regenerations) OR (“Wound Healing” OR “Healing, Wound” OR “Healings, Wound” OR “Wound Healings”) AND (“Plantago major” OR “Plantago officinarum”. Grey literature will not be included in the search.

### Procedure for study selection

At first, titles and abstracts will be examined by two reviewers (FCIC and PPA) and then selected according to the criteria for potentially eligible studies. Duplicated studies will be excluded from the search. Possible discrepancies between the two reviewer evaluations will be discussed and resolved or decided by a third member (APD). Subsequently, studies that were identified as eligible will be submitted to a text review performed by two reviewers (TP and JBC). Disagreements among the text reviewers will be resolved by a third member (ARSO-K). The final list of publications to be included in the systematic review will be decided in plenum. The following data will be extracted: title, author, year, journal, study type, wound kind, formulation type, treatment time, method, comparison groups, and outcome (Table 1). Remaining discrepancies will be resolved in agreement with a third author, and then, they will be revised in plenum. Data will be extracted to a Microsoft Office Excel document.

**Table 1:** Suggested data to be extracted from eligible papers

Publication details	Authors, year of publication, geographical location of study, funding sources.
Study design	Sample size, *p* values, methods to allocation, blinding of outcome assessment, number of the wound by animals, anatomical location of the wound, mechanism of producing the wound model. Concentration of *P. major* plant tissues used in each study.
Animal species	Rats, mice, rabbits
Animal characteristics	Age, weight, sex.
Duration of follow-up	The time of treatment and the wound closure.
Macroscopic analysis	Photo images, calculation of the retraction of the wound area until the wound closure.
Morphometric analysis	For example, reepithelialization and granulation-tissue formation, evaluated by histology; cell markers, e.g., cytokines, growth factors assessed by Immunohistochemical or ELISA analysis.

Statistical Analysis will be performed by HCO, ARSO-K, and MHM on data collected from January 2006 to January 2019.

### Criteria for inclusion and exclusion of studies

Studies selected for the systematic review must (a) be written in English; (b) use the animal model specifically in rats, mice, and rabbits; (c) include both genders; (d) and focus on acute or chronic cutaneous wound models. Studies must describe (a) the initial and final wound size, (b) the number of animals per group, (c) the time of treatment, (d) the present the concentration, (e) the formulation used in the treatment of the wound, (f) data about the wound-healing rate, (g) and cell markers that can be modulated by the therapeutic treatment, assessed by Morphometric analysis. Details are shown in Table 1. Studies using *P. major* mixed with another medicinal plant species will be excluded, as well as the ones based on in vitro experimentation.

### Intervention

The intervention group must include topical treatment with *P. major*. Treatment must describe formulation, concentration used, and initial and final size of the wound.

### Quality assessment

Studies will be evaluated by two independent reviewers with the quality evaluation instrument CAMARADES, a 10-item checklist in which one point is granted for each question [10]. The analysis of results will use the SYRCLE’s RoB [9] instrument, which consists of 10 “yes,” “no,” or “not clear” questions, indicating low, high, and not clear risk of bias, respectively.

### Comparators

Control group submitted to topical treatment with placebo/vehicle must be compared against the group receiving the analyzed intervention.

### Outcome

The outcome includes analysis of wound healing rates through macroscopic evaluation, photo images, or calculation of wound retraction area until wound closure.

### Data synthesis

The results of this systematic review will be presented in the narrative synthesis form. Data on animal species, wound type, treatment time, and results of interventions will be tabulated in order to support findings of the search. Random effects meta-analysis models will be employed to compare among groups, based on outcome variables. Studies included in the review must disclose sufficient high-quality data. Weighted mean differences among groups with their respective 95% confidence intervals and *p* values will be presented in forest plots. The presence of heterogeneity will be evaluated by *I*^2^ and chi-square statistical analyses. Funnel graphics will be constructed to evaluate publication bias.

## Discussion

To our knowledge, this is the first review to date evaluating effectiveness of *P. major*, a medicinal plant species, in wound treatments. This systematic review will be done solely for the English idiom due to financial constraints since it did not receive financial support. This is a limitation however attenuated by the fact that nowadays the vast majority scientific research is published in English. Results indicating efficacious and accelerated healing process using *P. major* topic treatment have been described in the literature. Even though efficiencies of this plant species in cutaneous healing process is not yet clear. Healing rates will be evaluated in cutaneous wounds that received *P. major* topical treatment compared with wounds that received placebo/vehicle treatment. *P. major* extract concentration, best response, time of use, and cell markers that can be modulated by the treatment are important variables to assess treatment effectiveness. Data on these variables might provide steady information; thus, they will be searched, compiled, and analyzed.

The results of this systematic review may contribute to the transferring of knowledge from *P. major* scientific research into clinical practice guidelines. Moreover, this systematic review will contribute with important information that could benefit future research efforts and potential applicability in humans.

## Data Availability

Not applicable

## Abbreviations

BVS: Virtual Health Library
Cab Direct: Centre for Agriculture and Bioscience International Bioscience International
CAMARADES: Collaborative Approach to Meta-Analysis of Animal Data from Experimental Stroke
ELISA: Enzyme-linked immunosorbent assay
Embase: Excerpta Medica Database
CINAHL: Cumulative Index to Nursing and Allied Health Literature
PROSPERO: International Prospective Register of Systematic Reviews
PubMed: Public/Publisher MEDLINE
Scopus: SciVerse Scopus
SYRCLE: Systematic Review Center for Laboratory animal Research
Web of Science: Medical Literature Analysis and Retrieval System
